# Dual Diagnosis of Thyroid Amyloidosis and Lipomatosis: An Uncommon Concurrence

**DOI:** 10.7759/cureus.104334

**Published:** 2026-02-26

**Authors:** Hyun Jin Kwon, Jacob Fiedler, Sohini Anand, Susana Vargas-Pinto, Paul Fiedler

**Affiliations:** 1 Pathology and Laboratory Medicine, Danbury Hospital, Danbury, USA; 2 Endocrinology Surgery, Danbury Hospital, Danbury, USA

**Keywords:** aa amyloid protein, amyloid goitre, congo red, fat-containing lesions of the thyroid, hyperthyroid, secondary amyloidosis, thyroid lipomatosis

## Abstract

Lipoid lesions of the thyroid are extremely rare. Adipose changes can be seen in both malignant (carcinomas) and benign conditions. The two most common benign entities are thyrolipoma and thyrolipomatosis, which have been reportedly associated with systemic amyloidosis. Radiologically, these lesions can mimic malignancy, especially when presenting as a biochemically active condition. We present a rare case of concurrent thyroid lipomatosis and serum amyloid A amyloidosis (AA) manifesting as goiter and subclinical hyperthyroidism. A 72-year-old male presented to Danbury Hospital with complaints of hoarseness of voice and reduced neck movement. His past medical history was significant for Crohn's disease, ankylosing spondylitis, monoclonal gammopathy of undetermined significance (MGUS), and AA amyloidosis involving bilateral kidneys with end-stage kidney disease (ESKD) status post renal transplantation. AA amyloidosis secondary to Crohn's disease led to bilateral kidney involvement and ESKD, requiring renal transplantation. Fine-needle aspiration of the left lower lobe in 2021 confirmed thyroid gland involvement, revealing atypical follicular cells with colloid present, classified as Bethesda Category III, with Congo red staining demonstrating amyloid deposition. Physical examination revealed severely enlarged thyroid glands causing compressive symptoms. Thyroid function tests indicated subclinical hyperthyroidism. Thyroid ultrasound revealed a right lobe measuring 9.0 × 5.5 × 4.8 cm and a left lobe measuring 9.0 × 5.5 × 4.3 cm, with a bilateral increase in size compared to imaging from two years prior. The left thyroid gland had a lower pole hypoechoic nodule measuring 1.0 × 1.0 × 0.8 cm, with mixed cystic and solid composition and smooth margins. No pathologically enlarged or morphologically abnormal cervical lymph nodes were identified on imaging. On follow-up, a total thyroidectomy with intraoperative nerve monitoring was performed. During follow-up in 2024, sonographically guided fine-needle aspiration of the left lower pole thyroid nodule was classified as unsatisfactory (Bethesda Category I), showing sheets of degenerated and anucleated squamous cells with debris, but no follicular cells. Thyroidectomy was subsequently performed due to symptomatic goiter with compressive symptoms. Gross examination of the specimen showed an abnormally enlarged thyroid gland with a diffusely yellowish cut surface. Hematoxylin and eosin (H&E) staining showed eosinophilic, amorphous, extracellular deposits, with diffuse infiltration of mature adipocytes in the thyroid stroma. A few entrapped residual thyroid follicles were observed. Congo red stain confirmed amyloid deposition, exhibiting apple-green birefringence under polarized light. Thyroid amyloid deposits are rare. Associated with chronic inflammatory conditions, including rheumatoid arthritis, ankylosing spondylitis, Crohn's disease, and familial Mediterranean fever (FMF). This case study illustrates the rare concurrence of thyroid lipomatosis and AA amyloidosis. It is thought that chronic hypoxia from AA amyloid-induced capillary damage: the driver of fibroblast-to-adipocyte metaplasia. Progression of this process resulted in diffuse replacement of the thyroid parenchyma with adipocytes and goitrous enlargement requiring resection.

## Introduction

In the normal thyroid gland, mature adipose tissue is generally restricted to pericapsular regions and areas surrounding blood vessels. This limited distribution reflects the gland's embryologic proximity to mesodermal structures of the neck, making the presence of fat around the anterior thyroid vasculature a relatively not unusual histologic finding. By contrast, mature adipose tissue embedded between the thyroid follicles of the central parenchyma is exceedingly rare [1,2]. Such a pattern has been documented only in a few unusual diagnoses, namely thyrolipoma and thyrolipomatosis. A review of the literature identified only 12 reported cases to date, of which merely six demonstrated concurrent adipose and amyloid deposition within the thyroid gland.

Histologically, thyrolipoma can be characterized as a thyroid follicular adenoma containing adipose tissue distinctively surrounded by a fibrous capsule. In contrast, thyrolipomatosis represents a diffuse proliferation of adipose tissue within the thyroid stroma and was first reported by Dhayagude in 1942 [1,3]. This diffuse pattern of fat distribution can be observed with focal microscopic amyloid deposition in association with systemic amyloidosis, medullary carcinoma, and primary amyloidosis of the thyroid gland [4]. The accumulation of adipose tissue together with amyloid causes diffuse enlargement of the thyroid gland, a condition referred to as amyloid goiter. Clinically, this can manifest as non-specific development of compressive symptoms, including dysphagia, dyspnea, and in advanced cases, progressive airway obstruction [5]. 

In this case report, we describe a rare case of thyroid lipomatosis with concurrent amyloidosis in a patient who initially presented with long-standing goiter secondary to amyloidosis, which subsequently was diagnosed as subclinical hyperthyroidism.

## Case presentation

A 72-year-old male patient presented to Danbury Hospital complaining of a diffusely enlarged thyroid gland since 2010. Ultrasound (US) of the thyroid gland at the time measured 6.2 × 2.7 × 2.6 cm on the right lobe and 6.2 × 2.9 × 2.8 cm on the left lobe, exhibiting diffuse enlargement and mild diffuse heterogeneity without nodules. Notably, this patient had a known history of Crohn's disease complicated by secondary serum amyloid A amyloidosis (AA) with marked bilateral renal involvement, ultimately progressing to end-stage renal disease, which necessitated kidney transplantation.

During 2011, sonographically guided core biopsy of the left thyroid lobe stroma highlighted prominent amyloidosis on Congo Red stain, in keeping with a diagnosis of amyloid goiter. In 2015, repeat US of the thyroid demonstrated a slight increase in size, with dimensions of the right thyroid lobe measuring 7 × 3.3 × 2.8 cm and the left lobe measuring 6.1 × 3.8 × 2.9 cm. Computed tomography (CT) of the neck without contrast was subsequently performed, exhibiting an abnormally enlarged thyroid gland with diffuse fatty infiltration and stable mild mass effect along the lateral margins of the trachea, slightly narrowing the transverse diameter but remaining widely patent.

The patient's thyroid continued to increase in size over the 14-year interval, measuring 9 × 5.5 × 4.8 cm and 9 × 5.5 × 4.3 cm on the right and left lobes, respectively, in 2024. At this stage, the patient's laboratory tests showed that he had subclinical hyperthyroidism and was treated with methimazole 5 mg daily. On physical exam, the thyroid gland was evidently enlarged, displacing the carotid vessels posterolaterally. The patient had a hoarse, crackly voice with significantly reduced neck movement. Free hormone levels were well controlled.

Given the progressive enlargement of the goiter, its associated subclinical hyperthyroidism, and emerging compressive symptoms, a total thyroidectomy with intraoperative nerve monitoring was recommended for this patient, and the post-operative course was largely uneventful. The patient was discharged on post-operative day three with levothyroxine 112 mcg and mycophenolic acid 180 mg daily. His chronic immunosuppressive regimen was also continued, including belatacept 250 mg, mycophenolate mofetil 360 mg, risankizumab 360 mg, along with apixaban 5 mg. 

Gross and histopathologic findings

Gross Examination 

The specimen was received in five parts with individual right and left thyroid lobes, each separate from its respective parathyroid glands and isthmus. The right lobe measured 8.5 × 6.5 × 3.6 cm and weighed 104 grams; the left lobe measured 9.8 × 5.7 × 5.5 cm and weighed 119 grams. Both lobes were markedly enlarged. The thyroid capsule was tan-pink in color, markedly disrupted and tattered on the right lobe, but predominantly intact on the left, where focal adhesions could be noted on the anterior surface. On serial sectioning, cut surfaces of both lobes revealed tan-yellow glistening rubbery parenchyma devoid of discrete lesions.

Microscopic Examination

**Figure 1:**
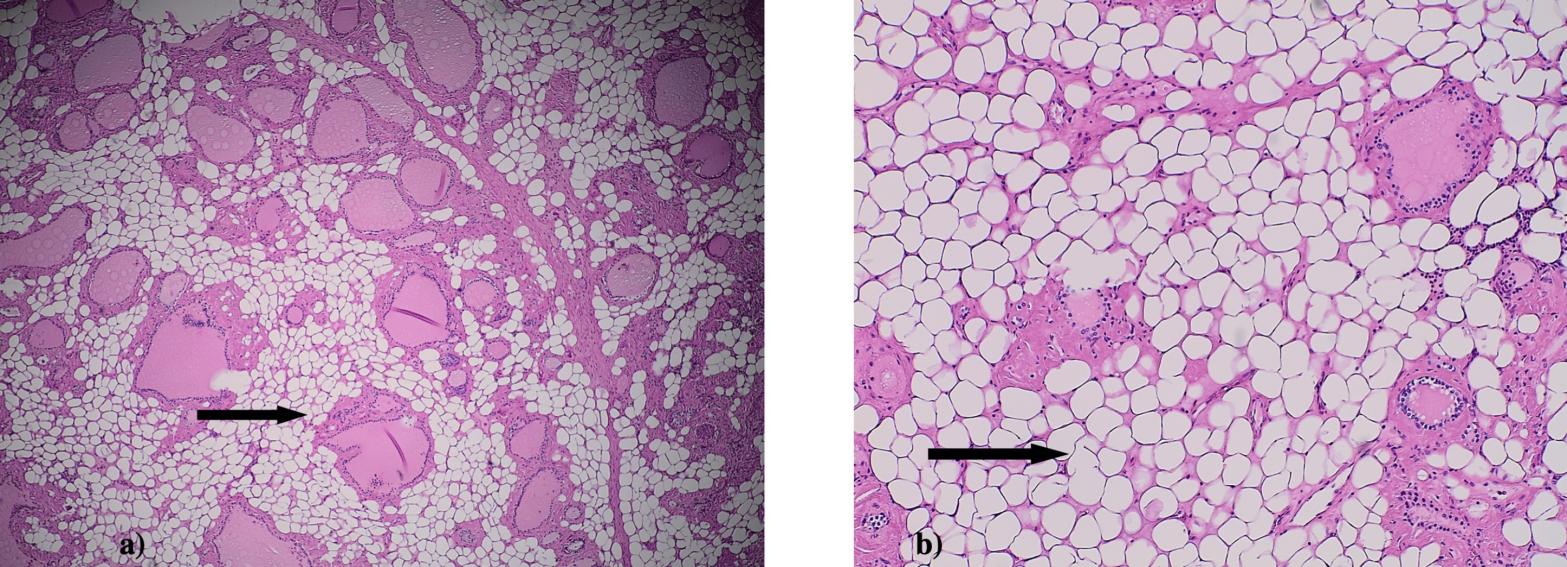
(a) and (b): Hematoxylin and eosin (H&E) stained sections at 4× and 10× magnification showing infiltration of the thyroid stroma by mature adipose tissue (indicated by black arrows).

**Figure 2:**
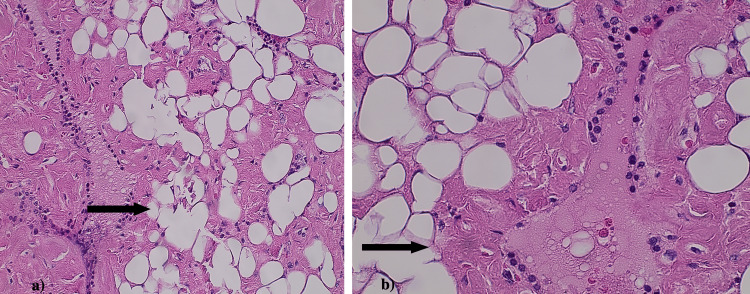
(a) and (b) Hematoxylin and eosin (H&E) stained sections at 20× and 40× magnification showing infiltration of the thyroid stroma by mature adipose tissue (indicated by black arrows).

Histopathology of multiple sections of thyroid tissue demonstrated diffuse infiltration of mature adipocytes within the perifollicular and perivascular stroma, accompanied by extracellular deposition of eosinophilic amorphous material, consistent with 60-70% amyloid (Figures 1, 2). Scattered, entrapped residual thyroid follicles with preserved architecture were noted in the background.

**Figure 3:**
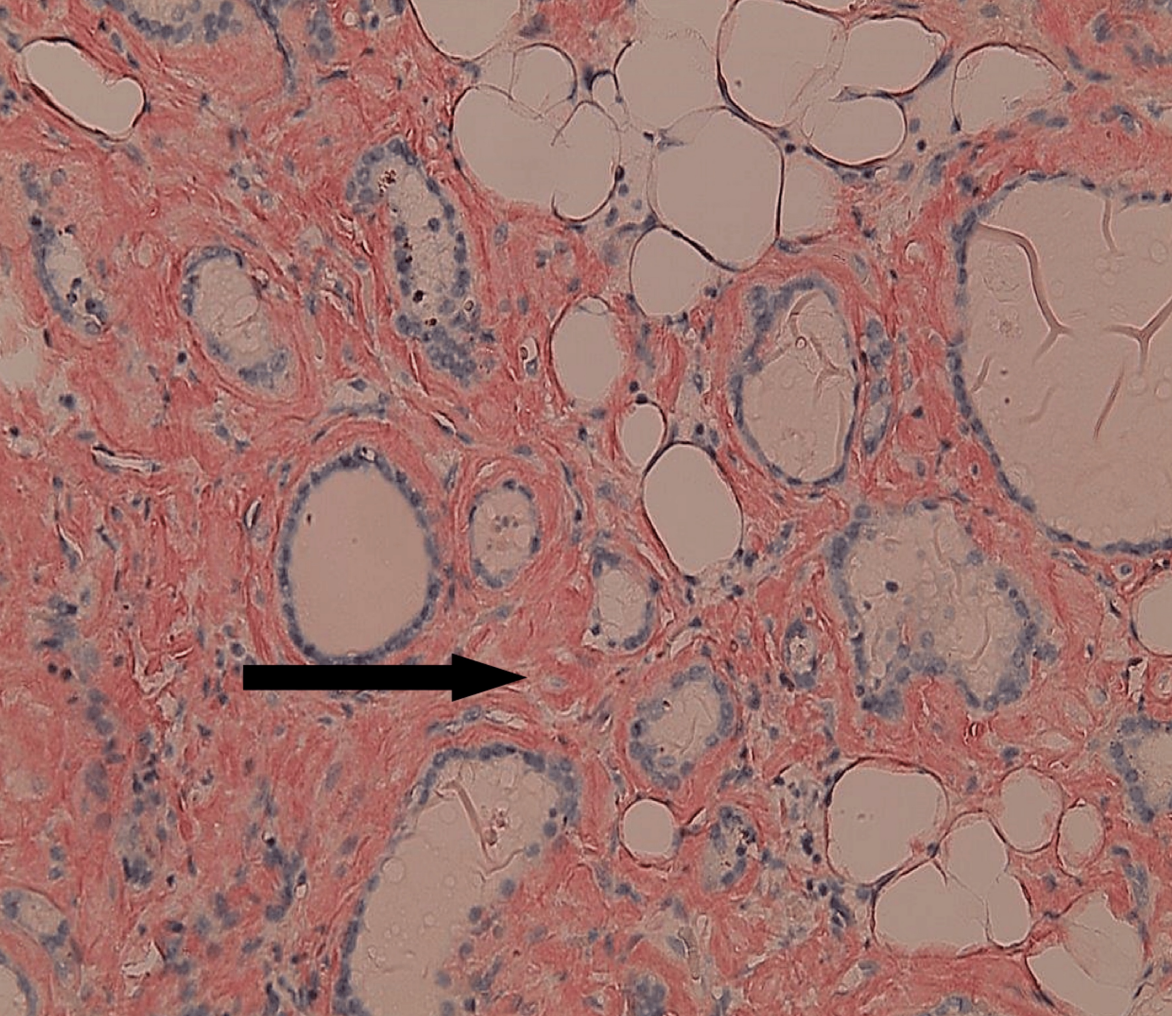
Congo red staining of interstitial amyloid protein (black arrow).

**Figure 4:**
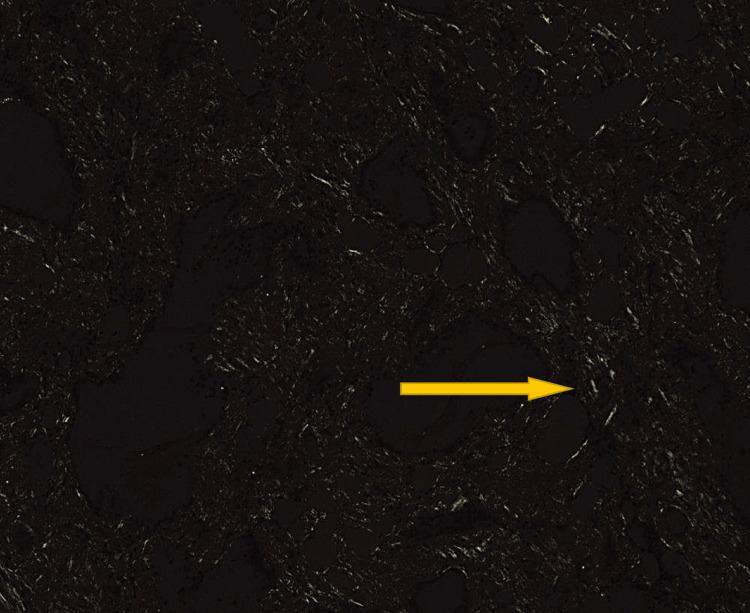
Congo red stain under polarized light microscopy demonstrating typical apple-green birefringence (yellow arrow).

Figure 3 demonstrates diffuse, intensely congophilic material deposited throughout the interstitium. The extent of deposition varies across examined fields; in some areas, the interstitium accounted for approximately 5% of the cross-sectional area, with the remaining 95% composed of adipose tissue, follicular epithelium, or colloid. In contrast, other regions, such as the area highlighted in Figure 3, showed interstitial involvement by amyloid comprising up to approximately 70% of the tissue. Overall, Congo red staining confirms extensive amyloid protein deposition, with characteristic apple-green birefringence observed under polarized light (Figures 3, 4). These findings supported a final diagnosis of diffuse lipomatosis of the thyroid gland with amyloid deposition.

## Discussion

Thyroid involvement by amyloidosis is relatively uncommon, and diffuse lipomatous change of the thyroid is even more rare. The concurrence of these two pathologies presents an exceedingly unique diagnosis with challenges that extend beyond routine thyroid disease evaluation. Although thyroid function tends to be preserved in the majority of cases, alterations in hormone levels have been described, with some patients presenting clinically with hypothyroidism or hyperthyroidism [5]. Our patient was initially diagnosed with subclinical hyperthyroidism, characterized by low thyroid-stimulating hormone (TSH) levels but normal circulating thyroid hormones (T3 and T4).

Thyroid involvement by amyloid alone, characterized by extracellular deposition of insoluble fibrillar proteins, occurs in approximately 50% of patients with primary amyloidosis and up to 80% of those with secondary amyloidosis [6,7]. AA amyloidosis was historically a frequent and devastating complication of chronic inflammatory diseases, particularly when untreated or poorly controlled; however, modern biologic therapies have substantially reduced prevalence [8]. In this present case, the patient's long-standing history of Crohn's disease likely created a chronic inflammatory state that promoted secondary AA amyloid infiltration. The accompanying deposition of adipose tissue, on the other hand, is thought to occur secondary to amyloid-induced capillary damage and subsequent tissue hypoxia. The resulting chronic hypoxic state likely fosters fibroblast-adipocyte metaplasia, leading progressively to the replacement of thyroid follicles with adipose tissue and diffuse goitrous enlargement [9]. However, it is important to note that alternative hypotheses have also been proposed. Some authors suggest that intraparenchymal adipose tissue may represent a developmental anomaly, potentially arising from adipose tissue entrapment during thyroid encapsulation, supported by the shared embryologic origin of the thyroid and thymus from the primitive foregut [10]. Another proposed mechanism is that the extensive lipid accumulation within follicular cells may reflect the metaplastic transformation of neoplastic thyrocytes [11].

With respect to clinical presentation, thyroid amyloidosis with lipomatosis most often manifests with symptoms related to progressive airway obstruction, including dysphagia, dyspnea, and changes to voice. These features, as in this present patient, tend to evolve insidiously over many years [12]. In this case, however, the most striking manifestation was end-stage renal failure, a well-recognised complication of systemic amyloidosis in conjunction with gradual thyroid enlargement [13].

Radiologic assessment posed additional diagnostic difficulties. The thyroid lobes were markedly enlarged, with a hypoechoic, partly cystic nodule and diffuse size progression over two years. Such sonographic features can readily suggest infiltrative malignancy. Gross examination compounded the challenge: the diffusely yellow cut surface of the resected gland was concerning for fat-rich or infiltrative processes, such as thyrolipomatosis, thyroid amyloidosis, metastatic disease, or, rarely, liposarcoma, emphasizing the need for histopathologic confirmation [14].

Despite the patient's known history of Crohn’s disease and systemic amyloidosis, cytologic evaluation remained challenging. In fact, fine-needle aspiration (FNA) was of limited utility: the first specimen demonstrated atypical follicular cells with amyloid deposition and was classified as Bethesda Category III, while the subsequent aspirate was unsatisfactory due to squamous debris and lack of follicular elements. These findings underscore a well-described limitation of FNA in amyloidosis, where amorphous deposits can mimic colloid or hyaline material and sampling is often inadequate. Lipomatous infiltration further reduces diagnostic yield because adipose tissue and degenerated material may predominate smears without providing representative follicular epithelium [15].

Similarly, lipomatous lesions of the thyroid are rare and can be mistaken for lipoma, liposarcoma, or adipose-rich variants of carcinoma. Histologically, distinguishing diffuse stromal adipose infiltration (thyrolipomatosis) from encapsulated thyrolipoma or from neoplastic mimics is essential [16]. Lipomatous change in particular represents a diagnostic pitfall, as focal fat accumulation may be dismissed as physiologic, while diffuse replacement can mimic aggressive tumor infiltration [17].

Ultimately, Congo red staining with demonstration of apple-green birefringence under polarized light remains the diagnostic gold standard for amyloidosis, while histologic identification of mature adipocytes infiltrating the thyroid stroma confirms lipomatosis [15]. Recognition of these entities is important to avoid unnecessary alarm for malignancy and to guide appropriate surgical decision-making, which, in most cases, is driven by compressive symptoms rather than oncologic urgency.

This case underscores how the co-existence of thyroid amyloidosis and lipomatosis can complicate clinical, radiologic, and cytologic evaluation. Awareness of these diagnostic pitfalls, particularly in patients with known systemic amyloidosis, is critical. More broadly, this dual pathology reinforces the need for a high index of suspicion, thorough histopathologic evaluation, and employing confirmatory special stains in patients with atypical thyroid presentations. In this patient, treatment of the hyperthyroid state with standard medical therapy did little to mitigate progressive thyroid enlargement, which was predicted to continue over time. As such, definitive management appears to be achieved only through total thyroidectomy, followed by thyroid hormone replacement therapy as required.

## Conclusions

This case represents an unusual occurrence of thyrolipomatosis with concurrent amyloidosis in a patient with a long-standing history of Crohn's disease. Among the few cases reported in the literature, the co-existence of these two pathologies is distinctly uncommon, especially with the additional finding of subclinical hyperthyroidism. Our case contributes to the limited body of evidence on this unique dual pathology and highlights the need for continued recognition of atypical presentations of thyroid enlargement.
